# Ketogenic diet as elective treatment in patients with drug-unresponsive hyperinsulinemic hypoglycemia caused by glucokinase mutations

**DOI:** 10.1186/s13023-021-02045-3

**Published:** 2021-10-11

**Authors:** Arianna Maiorana, Stefania Caviglia, Benedetta Greco, Paolo Alfieri, Francesca Cumbo, Carmen Campana, Silvia Maria Bernabei, Raffaella Cusmai, Antonella Mosca, Carlo Dionisi-Vici

**Affiliations:** 1 Division of Metabolism, Department of Pediatric Subspecialties, Ospedale Pediatrico Bambino Gesù, IRCSS, Piazza S. Onofrio 4, 00165 Rome, Italy; 2 Psychology Clinic Unit, Department of Neuroscience, Ospedale Pediatrico Bambino Gesù, IRCCS, Piazza S. Onofrio 4, 00165 Rome, Italy; 3 Child and Adolescent Psychiatric Unit, Department of Neuroscience, Ospedale Pediatrico Bambino Gesù, IRCCS, Piazza S. Onofrio 4, 00165 Rome, Italy; 4 Division of Artificial Nutrition, Ospedale Pediatrico Bambino Gesù, IRCCS, Piazza S. Onofrio 4, 00165 Rome, Italy; 5 Neurology Unit, Department of Neuroscience, Ospedale Pediatrico Bambino Gesù, IRCCS, Piazza S. Onofrio 4, 00165 Rome, Italy; 6 Department of Hepatology, Gastroenterology and Nutrition, Ospedale Pediatrico Bambino Gesù, IRCCS, Piazza S. Onofrio 4, 00165 Rome, Italy

**Keywords:** Hyperinsulinemic hypoglycemia, Ketogenic diet, Hypoglycemia, Neurodevelopment, Cognitive outcome, Epilepsy

## Abstract

**Background:**

Hyperinsulinemic hypoglycemia (HI) is the most frequent cause of recurrent hypoglycemia in children. Despite diagnostic and therapeutic advances, it remains an important cause of morbidity, leading to neurological complications, such as psychomotor retardation and epilepsy. Patients with diffuse drug-unresponsive HI manifest neurological impairment and neurobehavioral problems, even though surgically treated with a near-total pancreatectomy. Based on the analogies between HI and GLUT1 deficiency, both presenting with neuroglycopenia and lack of alternative cerebral energy sources, we administered a ketogenic diet (KD) in three drug-unresponsive GCK-HI patients with the aim of preserving neurodevelopment and avoiding the need of a near-total pancreatectomy. They presented recurrent symptomatic hypoglycemia, intellectual disability and refractory epilepsy. Patients were treated with classical KD for 79, 27 and 18 months, respectively.

**Results:**

All patients became asymptomatic in a few days and showed an important improvement of the alert state. Epilepsy disappeared and no appearance of novel hypoglycemic lesions was detected with a brain MRI. Cognitive and adaptive abilities rapidly improved and normalized. IQ rose significantly from 81 to 111 (*p* = 0.04) in patient 1, from 82 vs 95 (*p* = 0.04) in patient 2, from 60 to 90 (*p* = 0.04) in patient 3.

**Conclusions:**

We demonstrated the safety and efficacy of KD in the treatment of drug-unresponsive GCK-HI at a short and long-term. The neuroprotective effects of KD determined the recovery from epilepsy and intellectual disabilities and averted the need of a near-total pancreatectomy. All patients and their families reported an improvement of physical and psychosocial well-being, with a substantial improvement of their quality of life. These results might change the course and the quality of life of these patients and their families, having a relevant impact on human lives. Therefore, KD might be considered the elective treatment in unresponsive forms of GCK-HI.

**Supplementary Information:**

The online version contains supplementary material available at 10.1186/s13023-021-02045-3.

## Introduction

Hyperinsulinemic hypoglycemia (HI) is the most frequent cause of recurrent hypoglycemia in children [1] leading to a large percentage of neurological complications, such as psychomotor retardation and epilepsy [2–5]. HI is caused by uncontrolled or excessive insulin secretion for the prevailing glucose levels and it can be linked to genetic mutations in a number of genes involved in insulin secretion [6]. Due to the anabolic insulin effect, low/undetectable levels of ketones and fatty acids are characteristically recorded during hypoglycemia. The lack of alternative cerebral energy fuel supply is likely the main cause of the high rates of neurological complications in HI. Remarkably, the analysis of a large cohort of hypoglycemic patients comparing glycogen storage disease type 1 (GSD1), fatty acids oxidation defects (FAOD) and HI showed the highest prevalence of sequelae in FAOD and HI patients, in which hypoglycemia occurs without alternative energy sources [7]. Furthermore, in patients with GLUT1 deficiency, the impaired transport of glucose across the blood brain barrier induces neuroglycopenia without generation of other cerebral energy substrates, leading to epilepsy, developmental delay and movement disorders [8, 9]. In this rare disease, the ketogenic diet (KD) is the elective treatment, which effectively improves neurological outcomes by providing ketone bodies as an alternative cerebral energy source [8, 10]. Based on the similarities of brain metabolism perturbation shared by GLUT1 deficiency and HI (neuroglycopenia and lack of cerebral alternative fuels), we successfully utilized KD in a patient with severe drug-unresponsive HI with dominant mutation in glucokinase (*GCK*) gene (GCK-HI), presenting recurrent hypoglycemia, refractory epilepsy and mild intellectual disability [11]. Thereafter, we treated with KD two other drug-unresponsive GCK-HI patients, with the aim of preserving their neurodevelopment and avoiding the near-total pancreatectomy, an aggressive surgical procedure which does not guarantee a full recovery of disease [12] and is often associated with later appearance of diabetes and exocrine pancreatic failure [13–16]. Here we report the impact of the nutritional therapy with KD in the three patients with GCK-HI.

## Methods

### Patients

All patients presented a severe drug-unresponsive GCK-HI. Their main baseline characteristics are displayed in Table 1.

**Table 1:** Patients characteristics before starting ketogenic diet

	Patient 1	Patient 2	Patient 3
Age at diagnosis (year)	3	5^4/12^	3
Age at first observation (year)	9^8/12^	5^4/12^	6^6/12^
GCK mutation	p.Val455Met	p.Trp99Arg	p.Trp99Arg
HI therapy	DZX, OCT, NFD	DZX, OCT, SRL	DZX, OCT, SRL
Glucose/kg/min (mg)	5	20	8
Outcome	Symptomatic recurrent hypoglycemia Epilepsy Borderline IQ	Symptomatic recurrent hypoglycemia Vomiting Borderline IQ	Symptomatic recurrent hypoglycemia Epilepsy Mild ID
Anticonvulsivant therapy	Ethosuximide Valproate	.	Ethosuximide Clonazepam

*DZX* diazoxide, *OCT* octreotide, *NFD* nifedipine, *SRL* sirolimus, *IQ* intelligence quotient, *ID* intellectual disability

Patient 1 was previously described [11], presenting hypoglycemic seizures since the first 2 years of life. Despite high dose therapy and hyperglucidic diet, she had frequent hospitalizations for recurrent symptomatic hypoglycemia (0.5–1.6 mmol/L). She developed a refractory epilepsy (myoclonic and absence epilepsy) and an IQ lowering toward a borderline level (IQ 81), with a poor quality of life. In the attempt to avoid the near-total pancreatectomy which is required in unresponsive HI patients [17], at the age of 11.5 years we administered a KD with the aim to provide an alternative fuel for brain and to stem neurological damage. The new dietary regimen provided 85% of energy from lipids, 8% from proteins, and 7% from glucose in a normocaloric diet divided in 5 meals. The ratio of lipids to proteins plus carbohydrates was progressively increased every three months from 1.5:1 to 3:1 during the first year of KD in order to obtain a blood ketone bodies concentration close to 4 mmol/L, in analogy with GLUT1 deficiency recommendations [18].

Patient 2 presented with a severe persistent hypoglycemia requiring i.v. high glucose solution and i.v. glucagon for a high glucose demand. She was unresponsive to a high dose of diazoxide and octreotide, and presented food aversion and recurrent vomiting, therefore underwent gastrostomy for enteral nutrition. However, her glycemic control was still dependent on i.v. glucose and i.v. glucagon. At that time, given her recurrent vomiting and the need of i.v. glucose, KD was not considered as appropriate, rather sirolimus therapy was started, with initial efficacy [19]. However, after one year of treatment, her glycemic control deteriorated with recurrent symptomatic hypoglycemia and a decline of IQ to a borderline level (IQ from 113 WPPSI-III to 82 WISC-IV), hence sirolimus was discontinued and KD was finally started. Based on our previous experience, in this patient the ratio was more rapidly increased in four days from 1:1 to 3:1 to obtain an effective neuroprotective ketonemia (3–5 mmol/L).

Patient 3 presented with persistent symptomatic hypoglycemia, refractory epilepsy and a mild intellectual disability (IQ 60). In another center, trials with high dose of diazoxide, octreotide and sirolimus for 6 months were ineffective, therefore all therapies had been stopped. The child was addressed to our center, where KD was promptly started. The KD ratio was rapidly increased in 7 days from 1:1 to 2.7:1 to obtain an effective ketonemia (3–5 mmol/L).

The study was conducted in accordance with the Declaration of Helsinki Ethical standards and informed consent was obtained from parents. Ethical review and approval were waived for this study, according to our Ketogenic Diet Center clinical practices.

### Biochemical assays and instrumental procedures

Determination of blood ketones (Nova StatStrips® Glucose Ketone Meter, Nova Medical, Menarini Diagnostics, Florence, Italy) in the morning (after 10–12 h overnight fast), after lunch and after dinner was utilized as guidance to establish the adequate ratio of lipids to proteins plus carbohydrates. Auxological evaluation, hepatic and renal function, plasma glucose, insulin, FT4, TSH, IGF1, IGFBP3, plasma aminoacids, acylcarnitines, lactate, FFA, blood ketones and lipid profile, lipase, pancreatic amylase, electrolytes, blood gas analysis, along with calcium-phosphate homeostasis, renal tubular function, ECG, EEG were assessed every six months and abdomen ultrasound yearly. The follow-up included 96–120 h continuous glucose monitoring (CGM- ipro2, Medtronic; data analysis by MiniMed software, Medtronic, MiniMed, Northridge, CA, USA®). In patient 1 CGMS was performed simultaneously with EEG every three months for the first year. In the other two patients, CGMS was performed during the induction phase of KD, then respectively after two years and one year for the last time. Brain MRI was performed before and after KD.

### Neurocognitive evaluations

The Wechsler Intelligence Scales (WISC-III, WISC-IV, WPPSI-III, WAIS-IV) were serially administered every six months to evaluate cognitive skills.

Each child’s level of functioning, behavior and psychopathology was assessed every six months using the parental report of the Vineland Adaptive Behavior Scales (VABS) in survey form (using the interview version).

Parents also completed the parent form of the Child Behavior Checklist (CBCL), an instrument designed to evaluate the socio-emotional, psychopathological and behavioral functioning of children from the assessment of a variety of social and academic measurements.

Quality of life perception was assessed in children and their parents by using the PedsQL questionnaires. More information about psychological evaluation is described in the Additional file 1.

### Statistics

Statistical analyses were performed using SPSS 15.0 software (IBM Corporation, Somers, NY, USA). Standardized data about biochemical and auxological parameters were described by averages and SDs, and compared to the pre-KD results of each test. Wilcoxon test was used to evaluate the changes in the cognitive areas’ ordinal values from the start of KD onwards for each patient in a continuous distribution. Statistical significance was set for *p* values < 0.05.

## Results

Patient 1 was treated with KD for 79 months, patients 2 and 3 were treated for 27 and 18 months, respectively. Final KD ratios were 4:1, 3:1 and 2.7:1, respectively.

### Biochemical outcomes

The biochemical changes of each patient during KD are displayed in Table 2.

**Table 2:** Biochemical changes (mean ± SD) during ketogenic diet

	Patient 1	Patient 2	Patient 3	Mean
KD ketonemia (mmol/L)	3.1 ± 0.9	3.4 ± 0.9	4.6 ± 0.9	3.7 ± 0.8
Glycemia (mmol/L)	2.8 ± 0.4	2.2 ± 0.8	2.2 ± 0	2.4 ± 0.3
KD glycemia (mmol/L)	1.9 ± 0.2***	1.6 ± 0.3*	2.0 ± 0.4	1.8 ± 0.2
Insulin (μU/mL)	12.2 ± 5.1	18.2 ± 8.9	4.2 ± 0.7	11.5 ± 7.0
KD insulin (μU/mL)	10.1 ± 5.3	6.1 ± 4.9**	5.1 ± 2.1	7.1 ± 2.6
CGMS time in hypoglycemia (%)	32	68	50	50 ± 18
KD CGMS time in hypoglycemia (%)	37	100	100	79 ± 36.3

HbA1c n.v. 20–42 mmol/L; *NA* not available; *p < 0.05; **p < 0.01; ***p < 0.001

Optimal ketonemia between 3 and 5 mmol/L was reached in 1 year in patient 1. Based on the good results of safety and efficacy achieved [11], the effective ketonemia was reached more quickly, in 4 days in patient 2 and in 6 days in patient 3, in order to obtain the neuroprotective effect. Mean ketonemia was 3.7 ± 0.1 mmol/L.

During KD, the glycemic control of all patients worsened from a mean glycemia of 2.4 ± 0.3 to 1.8 ± 0.2 mmol/L (*p* = 0.07). CGMS performed during the induction phase of KD showed a rise of the time in hypoglycemia from a mean of 50 to 79% (*p* = 0.28). Despite persistence of hypoglycemia, related neurological symptoms disappeared in all patients.

Mean plasma insulin was reduced from pre-KD 11.5 ± 7.0 to post-KD 7.1 ± 2.6 μU/mL (*p* = 0.36) and significantly lowered only in patient 2 (*p* = 0.01).

Biochemical, endocrinological evaluations, ECG and abdomen ultrasound of all patients were normal, except detection of metabolic acidosis in patient 2, treated with sodium bicarbonate for 1 year, and a transient mild hyperuricemia in patient 1, treated with allopurinol.

### Auxological outcomes

Auxological parameters showed a normal linear growth, which corresponded to the target height. Weight changes were different between patients: after a rapid slimming during the first 6 months, patient 1 regained weight, developing a moderate obesity. Conversely, patient 2 lost some of her excess weight. Due to the high glucose demand, she became obese after diagnosis of HI, but as soon as KD was started she slimmed in 2 months showing a slight overweight. Patient 3 needed an increase of calories during the first 6 months because of an excessive weight loss, with a slight recovery thereafter (Additional file 2: Table 1). Overall, mean pre-KD BMI of patients was unchanged (pre-KD 20.4 ± 3.8 vs post-KD 21.9 ± 8.4, *p* = 0.79).

### Neurodevelopment

#### Epilepsy

EEG performed before KD in patients 1 and 3 showed generalized spike and wave discharges which disappeared after KD. Particularly, in patient 1 the first EEG was performed after 3 months (ratio 1.7:1) and in patient 3 after 7 days of KD (ratio 2.5:1), both resulting in normal readings. From the start of KD none of the patients showed epileptic crises nor absence epilepsy.

Patient 2 never manifested epilepsy, neither before nor after KD. However, during the induction phase, shifting from a KD ratio 2:1 to 3:1, her glycemia dropped sharply to 0.8–1.1 mmol/L and she presented hypoactivity/weakness for 24 h with a ketonemia of 3.5 mmol/L, but always maintained a normal level of consciousness. Intramuscular glucagon as bolus provided a glycemic rise with a secondary rebound of hypoglycemia, whereas continuous intravenous glucagon infusion was ineffective. Despite this transient hypoactivity, simultaneous EEG resulted normal. After 24 h, despite persistence of severe hypoglycemia, she presented normal consciousness and tone with a ketonemia between 5 and 7 mmol/L, therefore the ratio was reduced to 2.7:1. Overall, EEG performed after KD normalized in all patients despite persistence of hypoglycemia, as showed by CGMS performed concurrently.

#### Imaging procedures

Brain MRI was performed before and after KD. Patients 1 and 3 showed unchanged findings of small glyotic lesions of the white matter, likely outcomes of previous recurrent hypoglycemia. Patient 2 displayed a normal brain MRI before and after KD. In all patients, no abnormal restricted diffusion finding at MRI indicated no recent lesion.

#### Neurocognitive outcomes

Cognitive outcome improved in all patients (Table 3). In patient 1, despite the 1-year period required to reach the optimal value of ketonemia, neuropsychological and adaptive skills normalized within the first 6 months [11] and remained steady over the following 7 years. Normal values were reached in all neurocognitive areas, with a full-scale IQ 111 (*p* = 0.041) and a working memory index (WMI) 100 (WAIS; age 18y) at last follow-up.

**Table 3:** Outcomes after ketogenic diet

	Patient 1 Pre-KD > post-KD	Patient 2 Pre-KD > post-KD	Patient 3 Pre-KD > post-KD
Age at start KD (year)	11_6/12_	6_7/12_	6_6/12_
Age at last follow-up (year)	18^1/12^	8^10/12^	8
Follow-up (month)	79	27	18
KD ratio	4:1	3:1	2.7:1
Symptoms of hypoglycemia	Yes > No	Yes > No	Yes > No
Epilepsy	Yes > No	No > No	Yes > No
Epilepsy drugs	Yes > No	No > No	Yes > Yes
HI drugs	Yes > No	Yes > No	No > No
IQ	81 > 111*	82 > 95*	60 > 90*
AQ	94 > 93^†^/98^‡^	79 > 93^†^/89^‡^	76 > 81^†^/81^‡^
PedsQL	43 > 97	28 > 66	46 > 99

^†^VABS survey form; ^‡^VABS-II; *p < 0.05

**Fig. 1:**
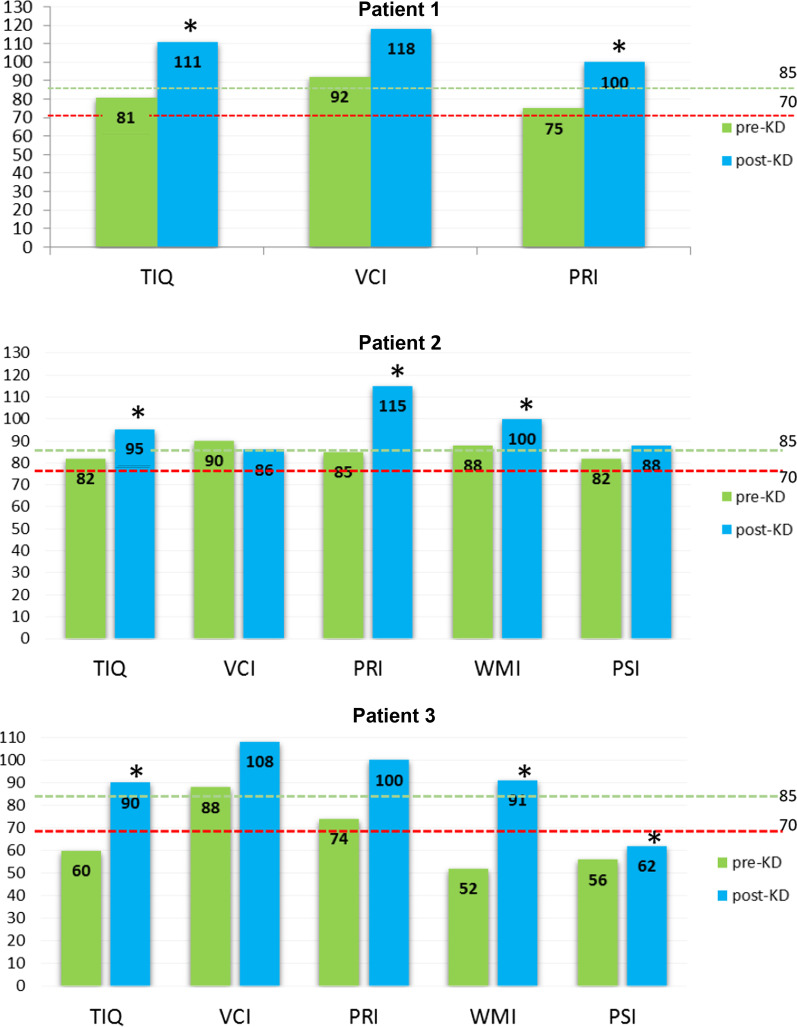
Cognitive skills evaluation from the start of ketogenic diet baseline to the last follow-up. All patients presented a significant improvement in IQ and in all indexes of Wechsler Intelligence Scales. Values > 85: normal; 85–71: borderline; 70–55: mild intellectual disability; *p < 0.05. *TIQ* total intelligence quotient, *VCI* verbal comprehension index, *PRI* perceptual reasoning index, *WMI* working memory index, *PSI* processing speed index

Patient 2 promptly recovered the cognitive deficit and her full-scale IQ stabilized at 93 (WISC-IV) over the following 21 months of treatment. Despite this general cognitive improvement, working memory index persisted near borderline (WMI 85), with a normal processing speed index (PSI 88) (WISC-IV; age 8y4m). However, at last follow-up, the working memory index improved (WMI 100, *p* = 0.042) (WISC-IV), despite a stable processing speed index 88 (*p* = 0.28) and a full scale IQ 95 (*p* = 0.039) (WISC-IV) (Fig. 1).

In patient 3, baseline neuropsychological assessments showed a mild intellectual disability, with worse abilities in working memory and processing speed skills. An impressive ameliorative trend over 18-month treatment follow-up was detected, with normalization of IQ and working memory skills: full-scale IQ 90 (*p* = 0.041); working memory index 91 (*p* = 0.033); processing speed index 62 (*p* = 0.038) (WISC-IV; age 8y) (Fig. 1).

#### Adaptive outcomes

With regards to adaptive functions, in view of the valuations with Vineland Survey Form, results showed a stable profile in patient 1 and in patient 3 with a small improvement. In patient 2 an ameliorating trend was noted over time, with better results concerning the Socialization domain (Socialization domain score 56; at last follow-up 95). The evaluation with Vineland II showed the same trend as with the Vineland Survey Form (Table 3).

#### Behavioral and Psychopathological outcomes

Concerning CBCL, parents’ reports showed heterogeneous results. In patient 1 normal results at baseline were obtained in the Internalizing Problems (IP 56), Externalizing Problems (EP 57) and the Total Problems Scales (TP 59). Since the KD was started, a worsening towards borderline scores have been detected in the Internalizing problems until the end of the follow-up. During all the evaluations the CBCL Youth Self-Report (YSR) was administered, with the finding of normal scores in each scale. In patient 2 Internalizing Problems were clinical (IP 72) at baseline, and improved through a borderline score (IP 61) at the last follow-up, Externalizing Problems maintained the normal range and Total Problems Scale normalized from baseline (TP 66) to last follow-up (TP 54). In patient 3 the scores remained within the normal range for each scale at all the evaluation times.

#### Quality of life outcomes

Quality of life assessments were performed before and after the start of KD in all patients. PedsQL Generic Score Scales assessing the parents' perception documented an improvement in the child’s quality of life, broadly encompassing physical, emotional, social, and school functioning domains (Table 3). In all patients the Self Report version confirmed the parents’ perception of an improvement in their own quality of life, in all the examined areas.

## Discussion

Despite diagnostic and therapeutic advances, HI remains an important cause of neurological disabilities [2–5]. Particularly, patients with diffuse and drug-unresponsive forms showed neurological impairment and neurobehavioral problems, even though surgically treated [16, 20]. However, a high percentage of patients who have undergone a near-total pancreatectomy have still required treatment for persistent hypoglycemia [12, 15], and appeared to be at risk of developing diabetes and exocrine pancreatic failure [13–16]. Among the unresponsive diffuse forms, GCK-HI is caused by dominant activating mutations of *GCK*, which overactivate the glucose-induced insulin secretion (GSIS) pathway, causing excessive insulin secretion, by lowering the glucose threshold for insulin release [21]. Some forms can be severe, requiring multiple pharmacological therapies until a near-total pancreatectomy, sometimes with persistence of hypoglycemia [22, 23]. The severity is variable depending upon the mutation in vitro. Our patients carried the mutations p.Val455Met (V455M) and p.Trp99Arg (W99R) which respectively have an estimated threshold for GSIS of 1.3–2.5 and 2.0–3.5 mmol/L, and are described to be diazoxide-responsive in vitro [23]. However, the spectrum of clinical phenotypes in vivo is very heterogeneous, regardless of the type of mutation [24], and in our patients it resulted in a severe phenotype, unresponsive to diazoxide and other pharmacological therapies.

With the aim to avoid the use of such an aggressive and often ineffective treatment as a near-total pancreatectomy, we administered KD to three patients with unresponsive GCK-HI, in order to provide an alternative energy source for the brain and to protect neurological development from the hypoglycemic damage.

As a matter of fact, it is known that hypoglycemic injury to the brain may cause intellectual disability since glucose is the main energy source available in patients with HI [4]. Specific deficits in memory and attention areas [25], visual and sensorimotor functions are underlined among neurocognitive complications compared to the general population [26]. Significant low scores in digit span subtest (related to working memory index in WISC-IV) have been observed in persistent HI compared to the standardization sample. These difficulties may appear as a consequence of hypoglycemic insult in childhood affecting the hippocampus, an important area for memory functions, as well as basal ganglia, parietal cortex, or posterior white matter, which are important areas for visual and sensorimotor functions [26]. The hypoglycemia-induced cellular injury includes excitatory neurotoxins, increased mitochondrial free radical generation and altered cerebral energetic characteristics [27]. Affecting neurometabolism through several pathways which counteract the hypoglycemia-induced damage, KD promotes a raise of neuronal ATP and mitochondrial biogenesis, and reduces free radical generation and neuronal excitability [28].

Our patients were treated with KD at different ratios, depending on the individual capacity to generate ketones in a range of 3–5 mmol/L. This value is similar to the recommended target level for GLUT1 deficiency [18], where blood β-hydroxybutyrate levels statistically correlated with seizure control [29]. We assumed that the protective effect from epilepsy at that range of ketonemia might be expression of a global neuroprotective effect and chose a ketone range of 3–5 mmol/L as a target safe level for short-term symptoms (symptomatic hypoglycemia, seizures) and long-term pathologies (intellectual disabilities, epilepsy). Particularly, in the induction phase of KD is very important to reach quickly the safe level to avoid symptomatic hypoglycemia. Indeed, maximum efficacy of KD is not achieved for several days or weeks after initiation, suggesting that adaptive metabolic and/or genetic “programs” underlie KD-induced protection and enhancement in energy production [28]. It means that in the induction phase the only direct protection from hypoglycemia are ketone bodies, as alternative cerebral fuel. Patient 2, who experienced worsening of hypoglycemia (because of low carbohydrate intake) and hypoactivity during the induction phase, had a normal EEG activity with a ketonemia on target, to witness of a direct neuroprotective effect of ketone bodies at short-term. However, in the induction phase of KD, hypoactivity has been reported in a few percentage of cases, independently from hypoglycemia [30]. Administration of glucose infusion would have suppressed ketogenesis. Therefore, the child was treated with single intramuscular glucagon and continuous intravenous glucagon infusion, the first giving a transient response, the latter being ineffective. The reduced intake of carbohydrates from KD reduces the hepatic glycogen content [31], indeed subjects under KD are at risk of hypoglycemia in the induction phase [30, 32, 33]. A single injection of glucagon is likely more efficacious to elicitate glycogenolysis from the poor hepatic glycogen reserves, than continuous glucagon administration, which might contribuite to their exhaustion, blunting its own response.

To reach an effective ketonemia, all patients needed a classical KD (ratio 4:1 for patient 1, ratio 3:1 for patient 2, ratio 2.7:1 for patient 3). The low ketogenesis rate was likely due to the inhibitory effect of hyperinsulinemia on lipolysis and ketogenesis pathways. Before starting KD, all patients presented symptomatic recurrent hypoglycemia and a decline of cognitive abilities, with borderline IQ and mild intellectual disability. One patient presented a rapid IQ reduction within a few months from the HI onset. Two patients developed a refractory epilepsy and the overall quality of life was poor. With the beginning of KD, although the glycemic levels further lowered (as shown by detection of persistent hypoglycemia at blood test, glucometer and CGMS evaluation), all patients became asymptomatic to hypoglycemia in few days and showed an important improvement of the alert state. Myoclonic crisis and absence epilepsy disappeared, EEG appeared normal, with absence of ictal EEG and of epileptic manifestations even during hypoglycemia. No appearance of novel hypoglycemic lesions was detected at brain MRI. The antiepileptic therapy was stopped in one patient and tapered in the other one. Hyperglycemic drugs were stopped in all patients.

Overall, cognitive abilities rapidly improved and normalized. Particularly, patients 1 and 2, who presented a borderline IQ, normalized it in 6 months; patient 3, who presented a mild intellectual disability, normalized his IQ in 18 months. However, HI patients can manifest milder specific neurocognitive problems, regardless the IQ level [26]. Under KD, those deficits tended to ameliorate slower at a long-term follow-up. Particularly, working memory index ameliorated significantly after 7 years in patient 1, in 2 years and 6 months in patient 2, in 1 year and 6 months in patient 3. Processing speed index normalized after 5 years in patient 1, after 2 years in patient 2, but appears still insufficient in patient 3. Cerebral regions responsible for higher cognitive functions such as attention, working memory, and executive functioning are at high glucose metabolism and their metabolic rate is particularly high from 4 to 10 years [34]. This might explain why those areas are particularly vulnerable in poorly controlled HI children and they are slower to respond to a low carbohydrate diet. In our patients KD was started after 6 years and all areas tended to improve, meaning that poor functioning is likely not permanent.

Adaptive abilities ameliorated in all patients, normalizing in patients 1 and 2, and improving up to a borderline level in patient 3. However, compared to cognitive abilities, adaptive scores resulted lower. A possible explanation can reside in the fact that adaptive abilities are determined by clinical interviews to parents. As a consequence, low score might be due to low parents expectations, because the answers to the interviews are based on parents perception. As usual for parents of children with chronic diseases, they might be overprotective, overcontrolling and intrusive, and interfere with child independence [26]; indeed both parents and children had a negative view of their condition, but parents showed more severity in the scores [35]. Alternatively, since IQ level improved more than adaptive functioning, neurobiological factors might be changed and have influenced the cognitive aspects rather than adaptive functioning, which consists of daily routine.

Regarding the behavioral and psychopathological aspects evaluated with CBCL, only patient 1 showed a slight worsening in scores of Internalizing problems, whereas the other patients ameliorated during KD likely becoming more self-confident, fearless of hypoglycemic symptoms and aware of their well-being.

All patients and their families reported an improvement of physical and psychosocial well-being, with a substantial improvement of their quality of life. Patients are living a normal life, attending school with good development, doing normal activities and socialization. Patient 1 obtained a grant and she is currently attending the University with good results.

KD affected neither auxological parameters nor endocrinological values, with mild and transient side effects of metabolic acidosis and hyperuricemia in some patients, in a safe profile.

Normoinsulinemic individuals are at risk of developing hypoglycemia in the induction phase of KD, in a proportion ranging from 1 to 28% [30, 32, 33]. In our HI patients, the glycemic control worsened in two out of three under KD. That was not related to the insulin levels, which resulted unchanged in two patients and reduced in the other one. GCK-HI patients are likely more at risk of hypoglycemia because the enzyme is costitutively activated for insulin secretion, while the carbohydrate intake is reduced under KD. Therefore, the worse glycemic control induced by KD was compensated by an optimal ketonemia which represented an effective alternative fuel for the brain, allowing seizure control and improving signs related to neuroglycopenia. However, the risk of sudden symptomatic hypoglycemia in case of non-compliance to KD needs to be emphasized to patients and families.

Other potential adverse effects of KD [36], such as gastrointestinal signs, electrolytes imbalance, carnitine deficiency, hyperlipidemia, pancreatitis, kidney stones, decreased growth, prolonged QT interval, osteopenia, were not recorded.

## Conclusions

We demonstrated the safety and efficacy of KD in the treatment of drug-unresponsive GCK-HI at a short and long-term. The neuroprotective effects of KD determined the recovery from epilepsy and intellectual disabilities and averted the need of a near-total pancreatectomy. Although limited by a small sample size for the rarity of disease, its promising results might change the course and the quality of life of these patients and their families, having a relevant impact on human lives. Therefore, KD might be considered as the elective treatment in the severe forms of GCK-HI.

## Supplementary Information

**Additional file 1.** Description of the tests used for evaluation of cognitive skills, adaptive abilities, children’s level of functioning, behavior and psychopathology, and quality of life.

**Additional file 2. Table 1:** Auxological parameters during ketogenic diet.

## Data Availability

The datasets used and /or analyzed during the current study are available from the corresponding author on reasonable request.

## Abbreviations

AQ: Adaptive quotient
CBCL: Child Behavior Checklist
GCK: Glucokinase
ECG: Electrocardiography
EEG: Electroencephalography
EP: Externalizing problems
FAOD: Fatty acids oxidation defects
GABA: Gamma aminobutyric acid
GCK: Glucokinase
CGMS: Continuous glucose monitoring system
GLUT1: Glucose transporter 1
GSD1: Glycogen storage disease type 1
GSIS: Glucose stimulated insulin secretion
HI: Hyperinsulinemic hypoglycemia
HRQOL: Health related quality of life
IP: Internalizing problems
IQ: Intelligence quotient
KATP channels: ATP-sensitive potassium channels
KD: Ketogenic diet
MRI: Magnetic resonance imaging
PedsQL questionnaires: Pediatric quality of life questionnaires
TP: Total problems
UCP2: Uncoupling protein 2
VABS: Vineland Adaptive Behavior Scales
WAIS: Wechsler Adult Intelligence Scale
WISC: Wechsler Intelligence Scales
WPPSI: Wechsler Preschool and Primary Scale of Intelligence
YSR: Youth Self-Report
