# Supplementary material for: Ketogenic diet as elective treatment in patients with drug-unresponsive hyperinsulinemic hypoglycemia caused by glucokinase mutations
Source: Orphanet J Rare Dis. 2021 Oct 11;16:424. doi: 10.1186/s13023-021-02045-3 (PMC8507241; doi:10.1186/s13023-021-02045-3)
Supplement: Supplementary file 1 — Additional file 1. Description of the tests used for evaluation of cognitive skills, adaptive abilities, children’s level of functioning, behavior and psychopathology, and quality of life. [file 13023_2021_2045_MOESM1_ESM.docx]

**Description of the tests used for evaluation of cognitive skills, adaptive abilities, children’s level of functioning, behavior and psychopathology, and quality of life**

**Wechsler Intelligence Scales**

The Wechsler Intelligence Scales (WISC-III, WISC-IV, WPPSI-III, WAIS-IV) [31-34] were serially administered every six months to evaluate cognitive skills. The WISC-III full -scale IQ consists of two indexes (verbal IQ and performance IQ with means of 100 and SD of 15; all of the three indexes consist of subtests with means of 10 and SD of 3. The WISC-IV full-scale IQ consists of four indexes (verbal reasoning, perceptual reasoning, working memory, and processing speed indexes) with means of 100 and a SD of 15. Each of the four indexes consists of subtests with means of 10 and a SD of 3. Lower scores indicate worse outcomes. The WAIS IV full-scale IQ consists of four indexes (verbal reasoning, perceptual reasoning, working memory and speed index) with means of 100 and a SD of 15. Each of the four indexes consists of subtests with means of 10 and a SD of 3. Lower scores indicate worse outcomes.

**Vineland Adaptive Behavior Scales**

Children’s level of functioning, behavior and psychopathology was assessed every six months using the parental report of the Vineland Adaptive Behavior Scales (VABS) in survey form [35] (using the interview version). The VABS is a semistructured parental interview that assesses multiple domains of children’s adaptive functioning, including communication, daily living skills and socialization. The score derived from this instrument has a mean of 100 and a SD of 15. Lower scores indicate worse outcomes. The latest and more updated version Vineland Adaptive Behavior Scales VABS-II [36] was administered for last two evaluations in all patients.

**Child Behavior Checklist**

Parents also completed the parent form of the Child Behavior Checklist (CBCL) [37], an instrument designed to evaluate the socio-emotional, psychopathological and behavioral functioning of children from the assessment of a variety of social and academic measurements. The two "broad band" scales “Internalizing Problems” (which sums the scores in anxious/depressed, withdrawn-depressed, and somatic complaints domains) and “Externalizing Problems” (which sums the score in Rule-breaking and Aggressive behavior), as well as the Total Problems Scale (which sums the scores of all the problem items) were considered. The score converts to a T-score for all scales: internalizing problems, externalizing problems and total (mean: 50; SD: 10; normal range T = 24 – 59, borderline T = 60 – 65, clinical T > 65). Higher scores indicate worse outcome.

**PedsQL**

PedsQL is a brief, standardized, generic assessment instrument that systematically assesses patients' and parents' perceptions by using the PedsQL questionnaires [38]. The PedsQL is based on a modular approach to measuring healthy related quality of life (HRQOL) and consists of a 15-item core measure of global HRQOL and eight supplemental modules assessing specific symptoms or treatment domains.

**References**

31. WISC-III, Wechsler Intelligence Scale for Children –Third edition, San Antonio, TX: Harcourt Assessment, 2002

32. WISC-IV, Wechsler Intelligence Scale for Children –Fourth edition, San Antonio, TX: Harcourt Assessment, 2003

33. WPPSI, Wechsler Preschool and Primary Scale of Intelligence – Third edition, San Antonio, TX: Harcourt Assessment, 2002

34. WAIS-IV, Wechsler Adult Intelligence Scale Fourth edition 2008 NCS Pearson

35. VABS, Vineland Adaptive Behavior Scales survey form, Sparrow S, Balla D, Cicchetti D. editors. Vineland adaptive behavior scales. Circle Pines, MN: American Guidance Service, 1984

36.VABS-II, Vineland Adaptive Behavior Scales – Second Edition, VABS, Balboni et al, 2016

37. CBCL, Child Behavior Checklist, Achenbach TM, Rescorla LA, editors. Manual for the ASEBA school-age forms and profiles. Burlington, VT: University of Vermont, Research Center for Children, Youth and Families, 2001

38. Varni JW, Gil KM, Schanberg LE. The PedsQL: measurement model for the pediatric quality of life inventory. Med Care 1999;37:126-39.
