# Supplementary material for: Ketogenic diet as elective treatment in patients with drug-unresponsive hyperinsulinemic hypoglycemia caused by glucokinase mutations
Source: Orphanet J Rare Dis. 2021 Oct 11;16:424. doi: 10.1186/s13023-021-02045-3 (PMC8507241; doi:10.1186/s13023-021-02045-3)
Supplement: Supplementary file 2 — Additional file 2. Table 1: Auxological parameters during ketogenic diet. [file 13023_2021_2045_MOESM2_ESM.docx]

**Table 1 Suppl. Auxological parameters during ketogenic diet**

|  | **Patient 1** | **Patient 2** | **Patient 3** |
| --- | --- | --- | --- |
| **Initial age** | 11 ^6/12^ | 6 ^7/12^ | 6 ^6/12^ |
| **Final age** | 18 ^1/12^ | 8 ^10/12^ | 8 |
| **BMI**  **(Z-score)** | 23  1.38 | 22  2.17 | 16  0.64 |
| **KD BMI**  **(Z-score)** | 31  1.7 | 20  1.26 | 14  -0.97 |
| **ibw**  **(%)** | 113 | 133 | 99 |
| **KD ibw**  **(%)** | 144 | 117 | 85 |
| **height**  **Z-score** | 0.68 | -1.15 | 1.5 |
| **KD height**  **Z-score** | 0.13 | -1.23 | 1.04 |
| **Target height**  **Z-score** | ±1.0 | 0/-2.5 | -1.0/2.0 |

BMI body mass index; ibw: ideal body weight;

ibw %: 90-109 normal weight, <90 underweight, 110-119 overweight,

120-139 mild obesity, 140-159 moderate obesity, >159 severe obesity
